# Metagenomic and Metabolomic Profiling Reveals the Impact of High‐Fat Diet on Malignant Pleural Effusion

**DOI:** 10.1111/1759-7714.70126

**Published:** 2025-07-21

**Authors:** Qing‐Yu Chen, Ming‐Ming Shao, Shu‐Feng Dong, Huan‐Zhong Shi, Feng‐Shuang Yi

**Affiliations:** ^1^ Department of Respiratory and Critical Care Medicine Beijing Institute of Respiratory Medicine and Beijing Chao‐Yang Hospital, Capital Medical University Beijing China; ^2^ Medical Research Center Beijing Institute of Respiratory Medicine and Beijing Chao‐Yang Hospital, Capital Medical University Beijing China

**Keywords:** high‐fat diet, malignant pleural effusion, metabolomics, metagenomics

## Abstract

**Background:**

Malignant pleural effusion (MPE) is a common complication in the advanced stage of cancer. High‐Fat Diet (HFD)‐induced obesity has become a common metabolic background in cancer patients. Recent studies have demonstrated that HFD induces gut dysbiosis, resulting in alterations in metabolites and immune responses. However, its role in MPE remains unclear.

**Methods:**

We established an MPE mouse model under both normal chow and HFD conditions. Metagenomic sequencing of fecal samples and untargeted metabolomics of plasma were performed to assess alterations in gut microbiota and systemic metabolites, respectively. Bioinformatic and statistical analyses were conducted to identify significant microbial taxa and metabolic pathways.

**Results:**

HFD‐fed mice exhibited increased pleural effusion. Metagenome data of the intestinal microbiome and metabolome profiles of plasma metabolites revealed key taxa—*Akkermansiaceae*, *Parabacteroides*, and *Muribaculaceae*—as well as significant metabolic pathways involved in sphingolipid metabolism, glycerophospholipid metabolism, and steroid hormone biosynthesis.

**Conclusion:**

These findings suggest that HFD may accelerate the MPE progression through modulation of gut microbiota and plasma metabolites, providing new strategies for prevention and treatment.

## Graphical abstract

The high‐fat diet modulates the gut microbiota and metabolic pathways in the mouse MPE model. Compared to mice fed a normal diet, those on a high‐fat diet exhibited increased abundance of *Parabacteroides* and decreased abundance of *Akkermansiaceae*. Key altered metabolic pathways in plasma included amino acid metabolism, xenobiotic metabolism via cytochrome P450 and choline metabolism.

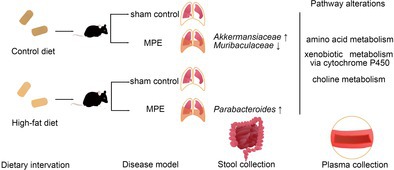

## 1 Introduction

Malignant pleural effusion (MPE) manifests in the advanced stages of malignancies, such as lung cancer, breast cancer, and lymphoma, affecting nearly 50% of lung cancer patients during treatment. MPE predicts a poor median survival of 4–7 months after the diagnosis, significantly shorter than that of patients without pleural effusion [1]. Thus, there is an urgent need for novel diagnostic biomarkers to facilitate the earlier detection and innovative therapeutic strategies to improve post‐diagnosis survival.

The rising prevalence of high‐fat (HFD) globally has become a concern, particularly due to its association with cancer progression. HFD is known to induce gut dysbiosis, increasing intestinal permeability and promoting the leakage of pro‐inflammatory factors, leading to a chronic inflammatory state that accelerates malignancy [2, 3, 4].

The gut‐lung axis has become a promising area, focusing on the interaction between gut microbiota and respiratory health. Many respiratory diseases have been linked with the gut microbiota, such as asthma, chronic obstructive pulmonary disease, respiratory infections, and cystic fibrosis [5, 6]. Microbial metabolites, including bile acid derivatives and short‐chain fatty acids, have been shown to affect the respiratory system. Moreover, there is an association between the gut microbiome and lung cancer progression [7, 8, 9, 10]. However, the relationship between gut microbiota, systemic metabolites, and pleural diseases, including MPE, remains largely unexplored.

In this study, we constructed an MPE model on the basis of an HFD model mouse. We characterized changes in the gut microbiome by metagenomic analysis and identified alterations in plasma metabolites by untargeted metabolomics. We hypothesize that HFD promotes MPE progression by inducing gut microbiota dysbiosis and reshaping microbial‐derived metabolic profiles, which in turn modulate the systemic and pleural immune microenvironment to favor malignant effusion formation and tumor immune evasion. By comparing MPE‐modeled mice to healthy controls, we identified specific plasma metabolites and explored their potential impact on gut microbiota. Then we analyzed the effects of HFD on the gut microbiome and plasma metabolites to estimate their possible contributions to MPE progression.

## 2 Method

### 2.1 Mouse Model

The mouse protocols in this study were conducted in accordance with the guidelines approved by the Institutional Animal Care and Use Committee of the Capital Medical University (AEEI‐2024‐043). C57BL/6J mice (Beijing Vital River Laboratory Animal Technology, China) were raised under the standard conditions (23°C–25°C, humidity 50%–60%, 12‐h light/dark) cycle.

#### 2.1.1 Malignant Pleural Effusion (MPE) Mouse Model

Mice were randomly selected to either the MPE group or the sham control group (SC). Lewis lung cells were cultured in DMEM with 10% fetal bovine serum and harvested in the logarithmic growth phase. Cell viability was verified to be > 95% using trypan blue exclusion before injection. In the MPE group, mice were anesthetized with intraperitoneal pentobarbital sodium (50 mg/kg) and injected intrapleurally with 1.5 × 10^5^ cells that were resuspended in 80 μL PBS. In the SC group, mice were injected intrapleurally with 80 μL PBS under the same anesthesia. Both groups were sacrificed by CO_2_ asphyxiation after 14 days.

#### 2.1.2 High Fat Diet (HFD) Mice Model

Three‐week‐old male C57BL/6J mice purchased from Beijing Vital River Laboratory Animal Technology Co. Ltd. were acclimated for 3 days after arrival and then randomly assigned to either a high‐fat diet (HFD) group or a normal diet (ND) group for dietary interventions. The HFD feed (D12492, Beijing KeAo Xieli Food Co. Ltd.) provides 60% energy from fat and the ND feed (#2112/2151, Beijing KeAo Xieli Food Co. Ltd.) provided 10% energy from fat. There are no restrictions on food and water intake, and the food and water were replaced weekly. Mice were fed the specific diets for 13 weeks to induce obesity and metabolic dysfunction in the HFD group, while the ND group received a normal diet for comparison. Body weight was monitored weekly.

### 2.2 Sample Collection

Fresh fecal pellets were collected directly from the anal orifice of live, unstressed mice. The mice were gently held over a sterile collection container, and fresh fecal pellets were allowed to fall naturally as the mouse defecates, and care was taken to prevent contamination with urine or environmental debris. Pellets were immediately snap‐frozen in liquid nitrogen and stored at −80°C for subsequent analysis. Plasma was collected by centrifugation of blood in EDTA anticoagulation tubes at 400 g. For each sample, 100 μL of plasma was mixed with 400 μL of a mixture of methanol and acetonitrile (2/1, vol/vol) and sonicated for 10 min on ice. Samples were stored overnight at −40°C. After centrifugation (12 000 rpm, 4°C, 20 min), 150 μL of supernatant from each sample was transferred to LC vials for LC–MS analysis. QC samples were prepared by mixing all samples in equal volumes.

### 2.3 DNA Isolation and Library Construction

Genomic DNA was extracted from the samples using the QIAamp Fast DNA Stool Mini Kit (Qiagen, Hilden, Germany) according to the manufacturer's protocol. The concentration of DNA was measured by a NanoDrop2000 spectrophotometer (Thermo Fisher Scientific, Waltham, MA, USA) and its integrity was verified by agarose gel electrophoresis. DNA samples were sonicated into fragmentation using the S220 Focused‐ultrasonicators (Covaris, USA), and the fragments were purified with Agencourt AMPure XP beads (Beckman Coulter Co., USA). Library construction was performed using the TruSeq Nano DNA LT Sample Preparation Kit (Illumina, San Diego, CA, USA) following the kit's instructions.

### 2.4 Metagenomic Sequencing

The libraries were sequenced on the Illumina NovaSeq 6000 platform, generating 150 bp paired‐end reads. Raw sequences in FASTQ format were processed using Trimmomatic (v0.36) (Trimmomatic:a flexible trimmer for Illumina sequence data) for trimming and quality filtering. Host contamination was removed by aligning the filtered reads to the host genome using Bowtie2 (v2.2.9), discarding all aligned reads.

### 2.5 Bioinformatic Analysis

Metagenomic assembly was performed using MEGAHIT (v1.1.2) (MEGAHIT: an ultra‐fast single‐node solution for large and complex metagenomics assembly via succinct de Bruijn graph, MEGAHIT v1.0: a fast and scalable metagenome assembler driven by advanced methodologies and community practices), generating scaffolds. Scaffold gaps were treated as breakpoints to create new contigs (Scaftigs), and only Scaftigs longer than 500 bp were retained. Open reading frame (ORF) prediction was carried out on the assembled scaffolds using Prodigal (v2.6.3) (Prodigal: prokaryotic gene recognition and translation initiation site identification), translating the ORFs into amino acid sequences. A non‐redundant gene set was constructed from all predicted genes using CD‐HIT (v4.5.7) with clustering parameters of 95% identity and 90% coverage. The longest gene in each cluster was selected as the representative sequence.

Clean reads from each sample were mapped to the non‐redundant gene set using Bowtie2 (v2.2.9) with 95% identity. Gene abundance for each sample was calculated accordingly. Taxonomic classification was performed using the NR database, and species abundance was determined based on gene abundance. Abundance profiles were created for each taxonomic level, including Domain, Kingdom, Phylum, Class, Order, Family, Genus, and Species.

The functional annotation of gene sets (amino acid sequences) was conducted using DIAMOND (v0.9.7) (Fast and sensitive protein alignment using DIAMOND) with an e‐value cutoff of 1 × 10^−5^ against databases such as NR, KEGG (From genomics to chemical genomics: new developments in KEGG, Data, information, knowledge and principle: back to metabolism in KEGG), eggnog (eggNOG v4.0: nested orthology inference across 3686 organisms), SWISS‐PROT, and GO. Carbohydrate‐active enzyme information was annotated by comparing the gene sets with the CAZy database (The Carbohydrate‐Active EnZymes database (CAZy): an expert resource for Glycogenomics) using HMMER (v3.1), and enzyme abundance was calculated from the corresponding gene abundance.

The raw LC–MS data were processed using Progenesis QI V2.3 software (Nonlinear Dynamics, Newcastle, UK), which performed baseline filtering, peak identification, integration, retention time correction, peak alignment, and normalization. Key parameters include: 5 ppm precursor tolerance, 10 ppm product tolerance, and 5% product ion threshold. Compound identification was based on the accurate mass‐to‐charge ratio (m/z), secondary fragment patterns, and isotope distributions, using databases such as the Human Metabolome Database (HMDB), Lipidmaps (V2.3), Metlin, and custom databases. The extracted data were further refined by removing peaks with missing values (ion intensity = 0) in more than 50% of the samples, replacing zero values with half the minimum observed value, and filtering based on compound quality. Compounds with scores below 36 out of 60 were considered imprecise and excluded. The positive and negative ion data were combined into a data matrix.

Taxonomic and functional abundance profiles were analyzed using R (v3.2.0). Principal Component Analysis (PCA), Principal Coordinates Analysis (PCoA), and Non‐metric Multidimensional Scaling (NMDS) were conducted to visualize data distribution. Statistical differences between groups were assessed using ANOVA, Kruskal–Wallis, *t*‐tests, or Wilcoxon tests as implemented in R. Linear Discriminant Analysis Effect Size (LEfSe) was used to identify significantly different features in taxonomic and functional abundance spectra.

Orthogonal Partial Least‐Squares‐Discriminant Analysis (OPLS‐DA) was used to discriminate metabolites between groups. To avoid overfitting, 7‐fold cross‐validation and 200 Response Permutation Testing (RPT) were used to assess the quality of the model. To rank the overall contribution of each variable to group differentiation, Variable Importance of Projection (VIP) values obtained from the OPLS‐DA model were used. A two‐tailed Student's *t*‐test was used to verify the significance of the difference between groups. Differential metabolites with VIP values greater than 1.0 and *p*‐values less than 0.05 were selected and further used for KEGG pathway enrichment analysis (http://www.genome.jp/kegg/). The bubble plot of KEGG pathway enrichment analysis of metabolites was visualized using Metorigin 2.0 (https://metorigin.met‐bioinformatics.cn/home/). R packages, including Hmisc (version 5.1–3) and igraph (version 1.4.1), were used to export files, which were subsequently visualized using Gephi (https://gephi.org/).

## 3 Results

### 3.1 Basic Phenotype of the HFD and the MPE Mouse Model

**FIGURE 1:**
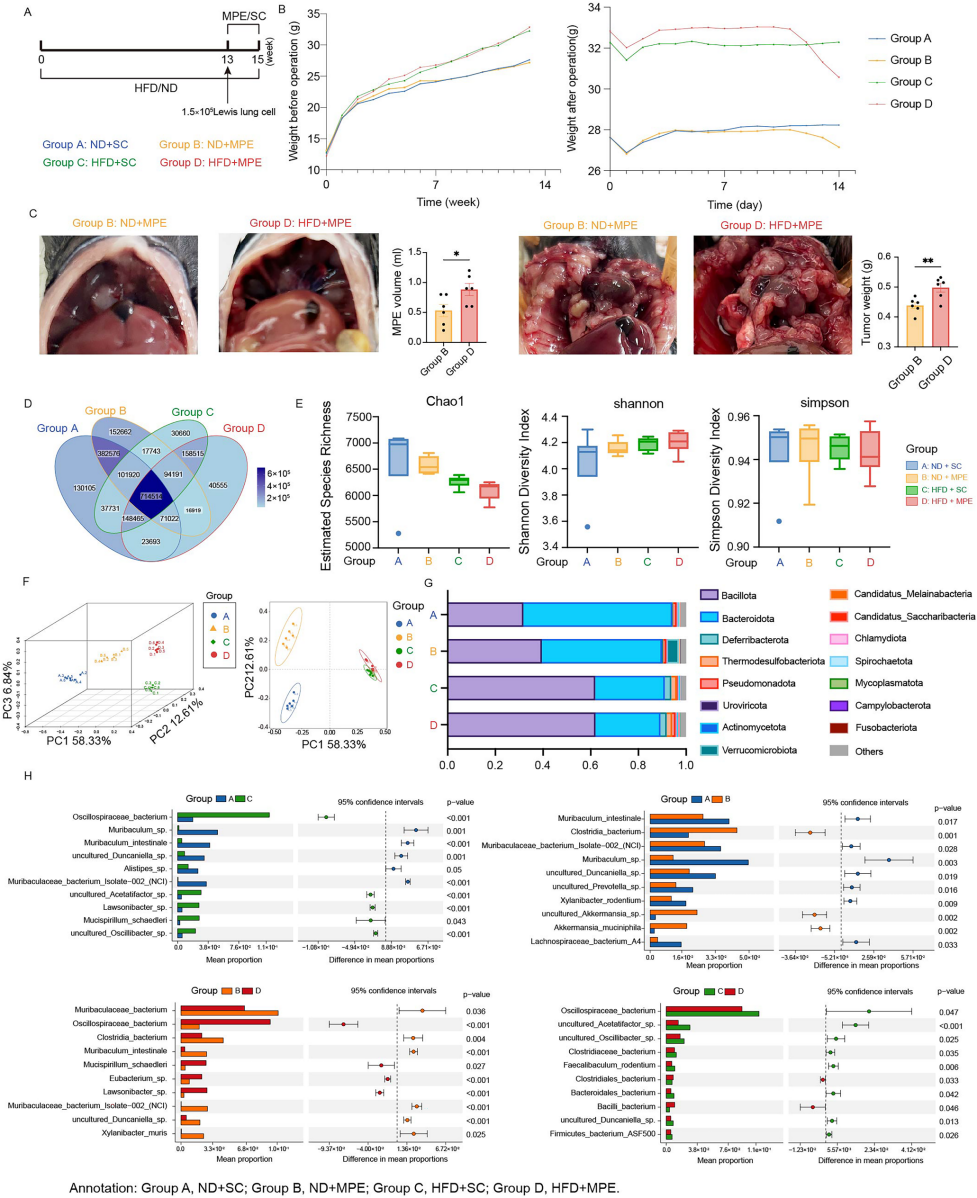
Basic characteristic and gut dysbiosis alterations of the High‐Fat Diet (HFD) and Malignant Pleural Effusion (MPE) modeled mice. (A) Schematic of experimental design illustrating group assignments for HFD and MPE models. HFD keeps for 13 weeks and followed by HFD and MPE for 2 weeks. 1.5 × 10^5^ Lewis lung cancer cells were injected intrapleurally. Mice were sacrificed on the 14th day after the injection. (B) (Left) Line chart of body weight during 13 weeks of HFD feeding before Lewis lung cell injection. (Right) Line chart of body weight during the MPE‐modeled phase. (C) Anatomical plots and bar plots of pleural effusion volume (Left) and tumor weight (Right) in MPE‐modeled mice. Data represent means (±SEM). *p < 0.05, with two‐tailed Student *t*‐test. (D) Venn diagram illustrating overlapping of taxa clusters among different groups. (E) Alpha‐diversity analysis (Chao1, Shannon, and Simpson) of gut microbiota using reads at the species level, each box represents the interquartile range (IQR) with the horizontal line denoting the median value. (F) 3D Principal coordinate analysis (PCoA) plot (Left) and the 2D PCoA plot (Right) based on the Bray Curtis distances. Each point represents the microbial composition of an individual sample, with colors corresponding to the specific groups. Ellipses denote 95% confidence intervals around group centroids. (G) Stacked Bar Chart of Taxonomic Composition. Each bar represents the average composition of microbial communities within a group, with colors indicating distinct phyla. (H) STAMP analysis of Taxonomic Composition using T‐test between either two groups. Bar plot represents abundance and scatter plot represents confidence intervals. Taxa with significant differences (*p* < 0.05) are filtered and the top 10 taxa by abundance are shown. HFD, high‐fat diet; MPE, malignant pleural effusion; ND, normal diet; SC, sham control.

Four groups of three‐week‐old mice (six mice per group) were assigned to different diets and treatments (Figure 1A). Groups A and B were fed the ND, while Groups C and D were given the HFD for 13 weeks. After 13 weeks of feeding, mice on the HFD (Groups C and D) exhibited significantly higher body weights (32.6 ± 0.3 g vs. 27.4 ± 0.6 g, Student *t‐*test, *p* < 0.0001) than those on the ND (Groups A and B; Figure 1B).

To induce the MPE model, Groups B and D received intrapleural injections of 1.5 × 10^5^ LLC cells suspended in 80 μL PBS, while Groups A and C were injected with 80 μL PBS as sham controls (SC). At the time of sacrifice (14 days post‐injection), Group B exhibited reduced body weight compared to Group A, and Group D had reduced weight compared to Group C (Figure 1B). Both MPE volume and tumor weight were significantly higher in Group D than in Group B (Figure 1C).

### 3.2 Gut Microbiome Composition and Diversity

To figure out the effect of HFD and MPE on the gut microbiota, metagenomic sequencing was performed on the fecal samples. The effective data volume for each sample ranged from 10.77 to 12.39 G. The N50 statistics for contigs were distributed between 2192 and 7016 bp. After deduplication, a non‐redundant gene catalog was constructed, containing 2 121 271 open reading frames. The annotation rate of the non‐redundant gene catalog was 96.63%.

To illustrate the distribution of clusters among groups, a Venn diagram was used to display the shared and unique clusters (Figure 1D). 714 514 clusters were shared among all groups. 16 919 were unique in the MPE but shared in the HFD and ND mice, while 158 515 clusters were unique in the HFD but shared in MPE and SC, indicating diet affects more in the diversity of microbial taxa than cancer. To address the difference in species diversity among the groups, we analyzed the alpha diversity metrics (Figure 1E). Mice fed on a HFD exhibited lower Chao1 and Abundance‐based Coverage Estimator values compared to those on a ND, while Shannon Indices and Simpson Indices were comparable between these groups. This indicates that though the dominant species was similar, HFD mice had fewer rare species. The Good's coverages of all four groups were over 99.997%.

To assess the differences in species composition, we performed a principal coordinate analysis (PCoA) analysis based on the Bray‐Curtis distances, demonstrating a clear group separation (Figure 1F). Taxonomic analysis further revealed shifts in relative abundance at the phylum level (Figure 1G). Consistent with previous studies, HFD mice exhibited a higher proportion of *Bacillota* and a lower proportion of *Bacteroidota* (also called *Firmicutes*) (Figure 1G). *Verrucomicrobiota*, which were further confirmed to be mainly driven by the increased abundance of *Akkermansiaceae*, were elevated in group B compared to A. This pattern was not observed in the group D vs. C comparison (Figure 1H). To identify the key microbial taxa, we calculated the Linear Discriminant Analysis (LDA) Effect Size (LEfSe) at the phylum, genus, and species levels. *Oscillospiraceae bacterium* and *Lawsonibacter* sp. exhibited significantly higher abundance in response to the high‐fat diet, regardless of the MPE, with *p* < 0.001 in both the C vs. A and D vs. B comparisons. In contrast, the abundance of *Muribaculum intestinale*, *Muribaculaceae* bacteriu Isolate‐002 (NCI) and uncultured *Duncaniella* sp. decreased under the high‐fat diet. Meanwhile, the effects of MPE on the microbiota in the HFD group were notably different from those in the ND group. In the ND group, the abundance of *Muribaculum intestinale,* *Muribaculaceae* bacterium Isolated‐002 (NCI), *Muribaculum* sp., uncultured *Duncaniella* sp., uncultured *Prevotella* sp., *Xylanibacter rodentium* and *Lachnospiraceae* bacterium A4 decreased under MPE, while Closteridia bacterium, uncultured *Akkermansia* sp. and *Akkermansia muciniphila* increased. In the HFD group, *Oscillospiraceae* bacterium, uncultured *Acetatifactor* sp., uncultured *Oscillibacter* sp., *Clostridiceae* bacterium, *Faecalibaculum rodentium*, *Bacteroidales* bacterium, uncultured *Duncaniella* sp. and *Firmicutes* bacterium ASF500 decreased, while *Clostridiales* bacterium and *Bailli* bacterium increased.

**FIGURE 2:**
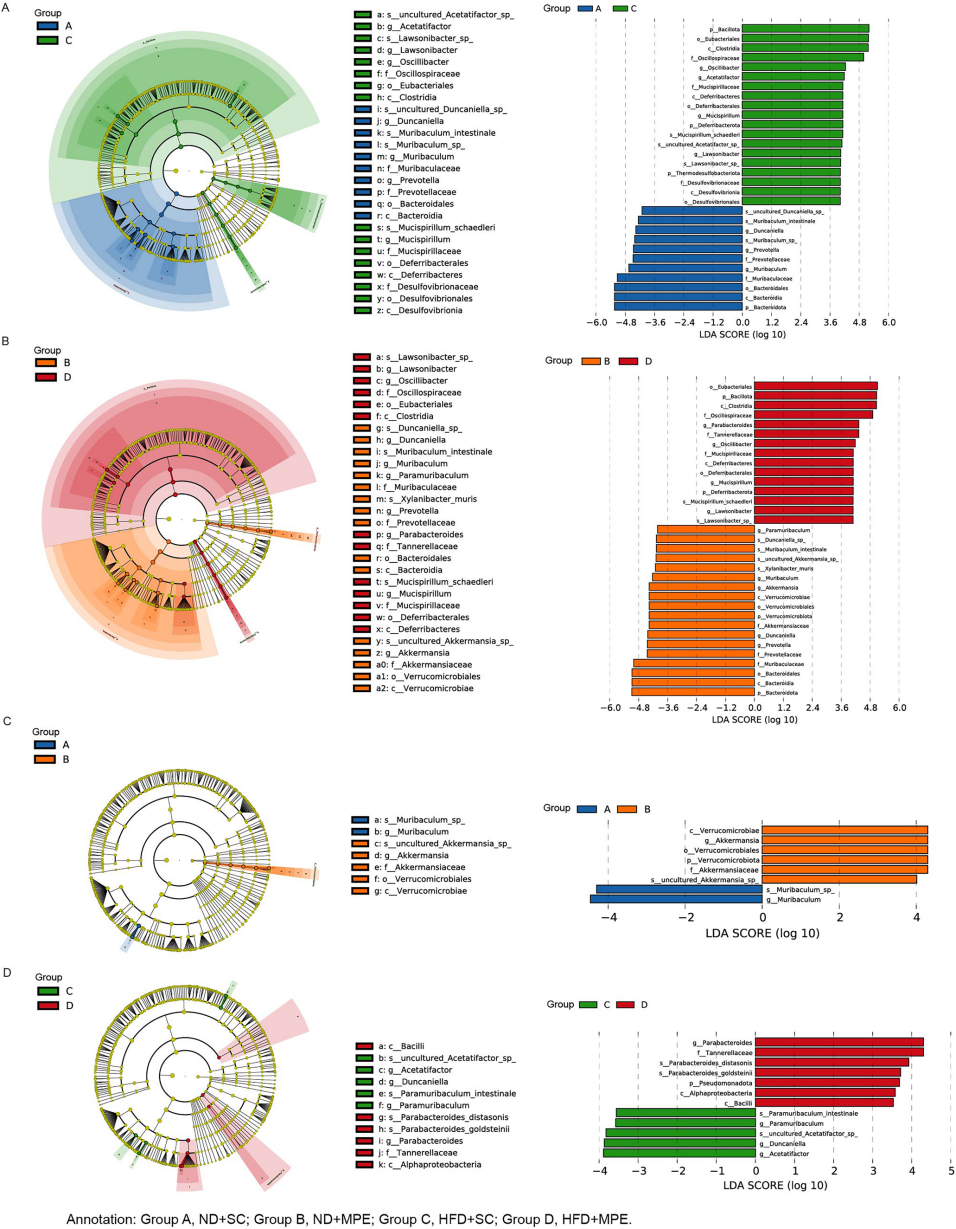
Whole levels analysis of gut dysbiosis alterations in HFD‐fed mice and MPE‐modeled mice. (A–D) (Left) Cladogram analyzing differential species annotation. From inner to outer layers, the nodes represent taxonomic levels: Kingdom, phylum, class, order, family, genus, species. Species names marked with letters are detailed in the legend. (Right) LEfSe analysis (Linear Discriminant Analysis Effect Size) Bar plot analyzing differential species contribution. Prefixes like k, p, c, o, f, g, and s before species names correspond to taxonomic ranks (kingdom, phylum, class, order, family, genus, species). HFD, high‐fat diet; MPE, malignant pleural effusion; ND, normal diet; SC, sham control.

To acquire an overview of the different metagenome profiles, a cladogram was drawn to compare the taxa at each level (Figure 2A–D). The comparisons between ND and HFD show more difference than the comparisons between SC and MPE, which means HFD does more effect on the intestinal flora than MPE did. In the HFD group, *Parabacteroides distasonis* and *Parabacteroides goldsteinii* were elevated, and *Acetatifactor*, *Duncaniella*, and *Paramuribaculum* were decreased in response to MPE.

**FIGURE 3:**
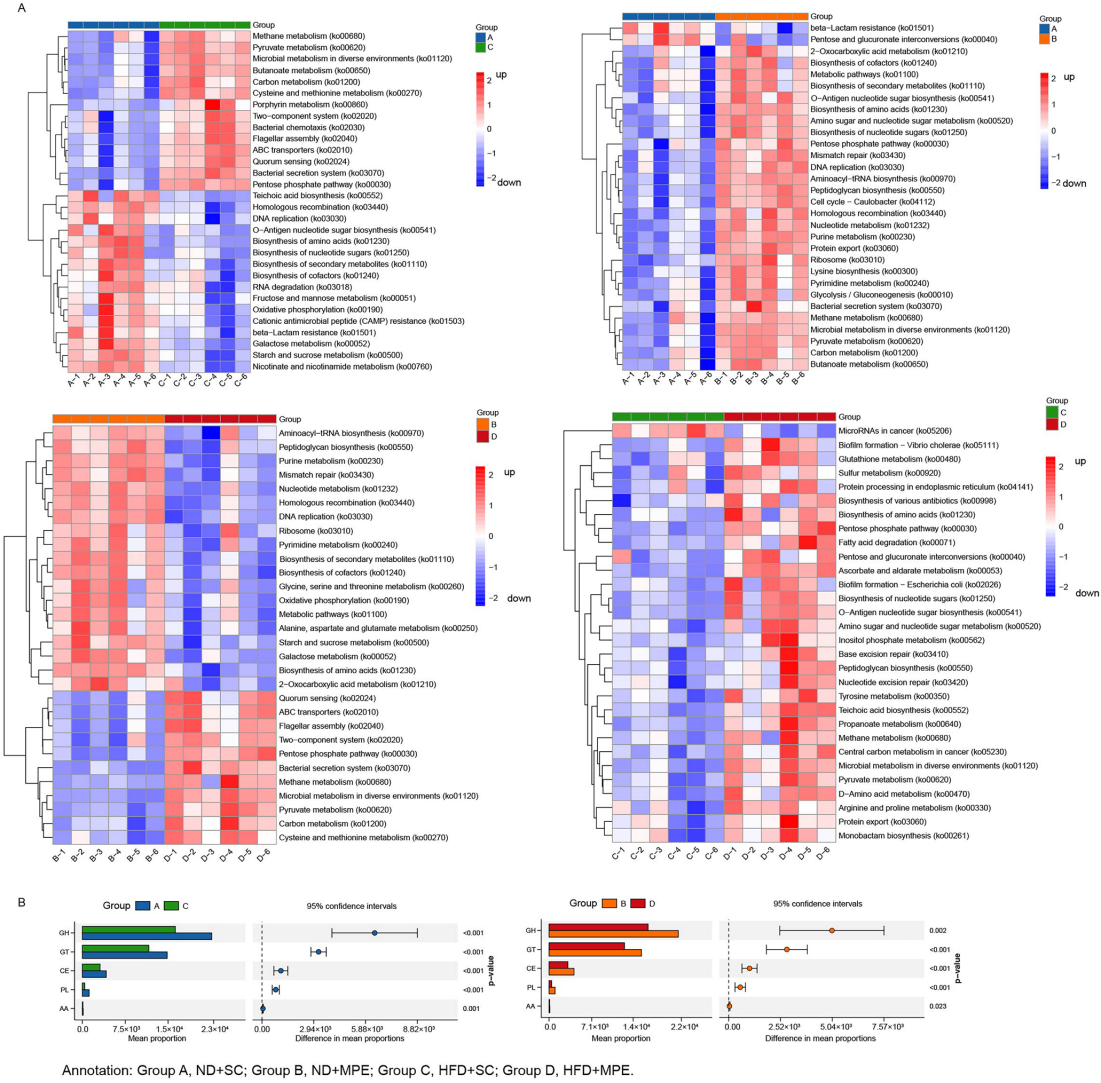
Kyoto encyclopedia of genes and genomes (KEGG) and carbohydrate‐active enzymes database (CAZy) analysis of gut microbiome on metagenomic sequencing. (A) Heatmap showing the relative enrichment of each sample in the Top 30 altered KEGG pathways based on the metagenomic analysis. (B) Comparative analysis of microbial functional genes based on the CAZy analysis. Bar charts (Left) represents the abundance and corresponded scatter plot (Right) represents the confidence interval. AA, auxiliary activities; CE, carbohydrate esterases; GH, glycoside hydrolases; GT, glycosyl transferases; HFD, high‐fat diet; MPE, malignant pleural effusion; ND, normal diet; PL, polysaccharide lyases; SC, sham control.

As the alteration of the intestinal flora causes the changes in the metabolome, we analyzed the KEGG enrichment based on the metagenomic analysis to shed light on the function of the taxa (Figure 3A). Pyruvate metabolism, methane metabolism, bacterial secretion system, carbon metabolism, cysteine and methionine metabolism, two‐component system, pentose phosphate pathway, and ABC transporters were elevated, while biosynthesis of secondary metabolites, biosynthesis of cofactors, biosynthesis of amino acids, and DNA replication were downregulated due to HFD. For response to the MPE, O‐Antigen nucleotide sugar biosynthesis, amino sugar and nucleotide sugar metabolism, pentose phosphate pathway, methane metabolism, and pyruvate metabolism were upregulated. But in the HFD group, MPE upregulated the tyrosine metabolism and arginine and proline metabolism, which differed from the upregulation of lysine biosynthesis in the ND group.

Given that microbial carbohydrates contribute to the tumor microenvironment, we analyzed the Carbohydrate‐Active Enzyme (CAZy) profiles of the intestinal flora (Figure 3B). Similar results were observed in both C vs. A and D vs. B comparisons. Enzymes involved in glycoside hydrolases, glycosyltransferases, carbohydrate esterases, polysaccharide lyases, and auxiliary activities were downregulated.

**FIGURE 4:**
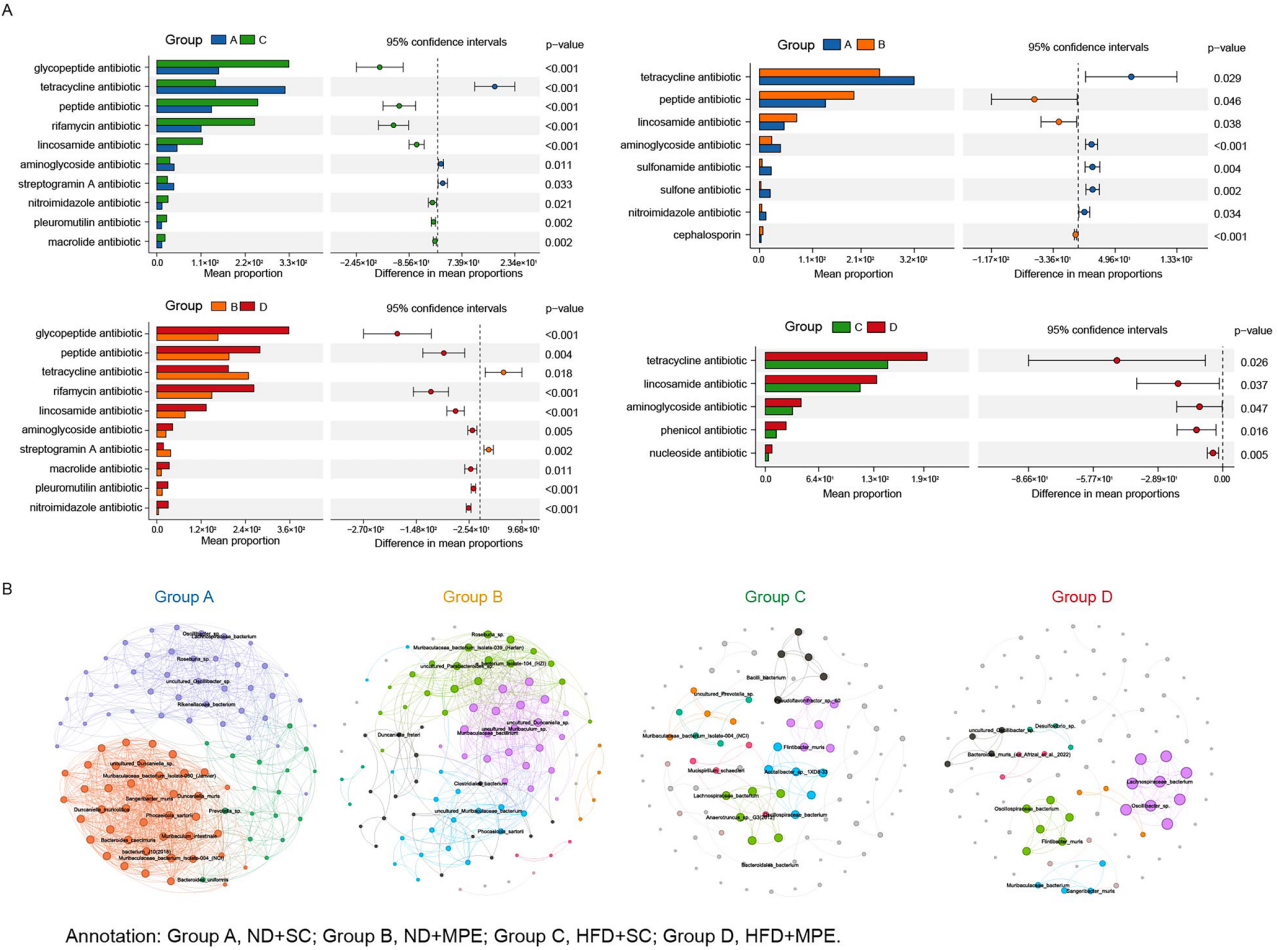
Comprehensive antibiotic research database (CARD) and correlation network analysis of gut microbiome on metagenomic sequencing. (A) Comparative analysis of microbial functional genes between either two groups for a single variant based on the CARD analysis. Bar charts (Left) represents the abundance and corresponded scatter plot (Right) represents the confidence interval. (B) Correlation network of the taxa analyzed at the species levels. Nodes represent individual taxa at the species level, with node size proportional to their relative abundance, node color indicating the different modules. Edges indicate significant correlations (*p* < 0.05, |r| > 0.8, Spearman's test). HFD, high‐fat diet; MPE, malignant pleural effusion; ND, normal diet; SC, sham control.

To investigate the possible antibiotic resistance which will be acquired by the intestinal flora, we performed the analysis based on the Comprehensive Antibiotic Resistance Database (Figure 4A). C vs. A and D vs. B showed a similarly elevated abundancy of antibiotic resistance. B vs. A and C vs. D showed a different tetracycline antibiotic; this indicated the different response under the HFD.

To explore the possible interactions among the microbial taxa, correlation network analysis was performed among the taxa with the Top 100 abundancy at the species level in each group (Figure 4B). In the MPE mice, the networks revealed fewer nodes and edges as well as lower density compared to the SC group. Less connected microbial network and clusters were associated compared to the ND group, indicating reduced microbial interactions under HFD conditions. The HFD + MPE mice network exhibits the fewest interactions.

We assumed that alterations in the intestinal microbiota induced by the HFD drive different activations of metabolic pathways in response to MPE, resulting in subsequent changes in the metabolite profiles.

### 3.3 Plasma Metabolome and Alterations

**FIGURE 5:**
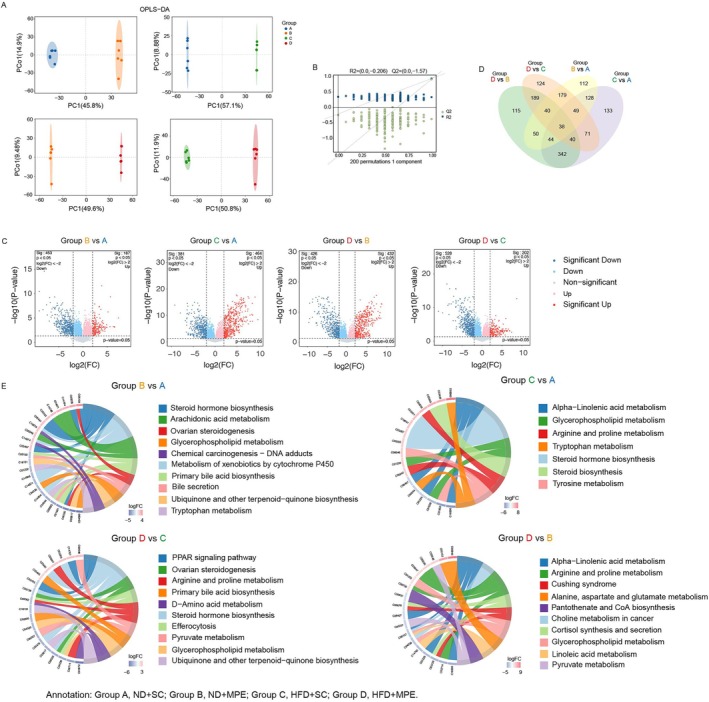
Metabolite profiles in HFD‐fed mice and MPE‐modeled mice. (A) Orthogonal projections to latent structures‐discriminant analysis (OPLS‐DA) score plot between two groups. (B) Permutation Plot including all metabolic samples evaluating the effectiveness of the OPLS model. (C) Volcano plot visualizing the differential metabolites based on *p*‐values and fold change (FC) values. Red dots represent significantly upregulated metabolites in the experimental group, while blue dots indicate significantly downregulated metabolites. Gray dots denote non‐significant metabolites. (D) Venn diagram illustrating differential metabolites in four comparable groups. (E) Pathway enrichment analysis represented by a chord diagram. Chord diagram illustrating the relationship between differentially expressed metabolic KEGG compounds and enriched Reactome pathways. KEGG compounds are represented on the left side with colors showing the up and down regulation. HFD, high‐fat diet; MPE, malignant pleural effusion; ND, normal diet; SC, sham control.

Gut microbiome could produce the metabolite that will enter the blood stream to affect the host environment; meanwhile, cancer in the MPE will alter the metabolites in the blood. To study how metabolite profiles changed when having an HFD diet and bearing MPE in the plasma, we applied LC–MS/MS technique on the mouse plasma among those four groups. 1847 and 2433 peak features were identified in positive (ES+) and negative (ES−) ion modes, respectively. A total of 4280 metabolites were detected in the plasma samples, of which 40.02% were lipids and lipid‐like molecules. We applied a log_2_ transformation to the data after removing zero values to reflect the variance in between and within the group. We applied Orthogonal Projections to Latent Structures‐Discriminant Analysis (OPLS‐DA) to the metabolite profiles to reduce the background noise and outfit the difference between groups (Figure 5A). After validation of 7‐fold cross validation and 200 response permutation testing (RPT), OPLS‐DA showed no overfitting (Figure 5B). Variable importance in projection (VIP) was acquired based on the OPLS‐DA model. To figure out the possible alteration in the plasma metabolites, comparisons based on one alteration in the invention of experimental design were performed between Group C and Group A (C vs. A), between Group D and Group B (D vs. B) (for the alteration of HFD) as well as between Group B and Group A (B vs. A), between Group D and Group C (D vs. C) (for the alteration of MPE). Those metabolites that met the criteria of VIP > 1, *p*‐value < 0.05, and FC ≥ 4 or FC ≤ 1/4 were selected (Figure 5C). Thirty eight metabolites were shared in the four compared groups, suggesting their potential involvement in both the HFD‐induced gut microbiome change and cancer progression. One hundred and seventy nine metabolites were found in both the B vs. A and D vs. C groups, indicating that these changes were possibly affected by MPE rather than the HFD. Three hundred and forty two metabolites were common between the C vs. A and D vs. B groups, suggesting the change was driven by HFD rather than MPE (Figure 5D).

To understand the change of the biological process under the common situation and under the overlay effects, we performed the pathway enrichment based on the metabolic KEGG compounds and enriched Reactome pathways (Figure 5E). The common enrichment which may be caused by either MPE or HFD were steroid biosynthesis and glycerophospholipid metabolism. Both B vs. A group and D vs. C group have an enrichment in ubiquinone and other terpenoid‐quinone biosynthesis, which means this pathway altered under the MPE condition whether HFD or ND were taken. Compared to the B vs. A group, the comparison of D vs. C group reflects the specific change of MPE under the HFD, indicating that PPAR signaling pathway was the specific MPE metabolic pathway under the HFD. Both C vs. A and D vs. B had enrichment on the alpha‐linolenic acid metabolism indicating the possible alteration in the change because of Warburg effects of the cancer cells.

**FIGURE 6:**
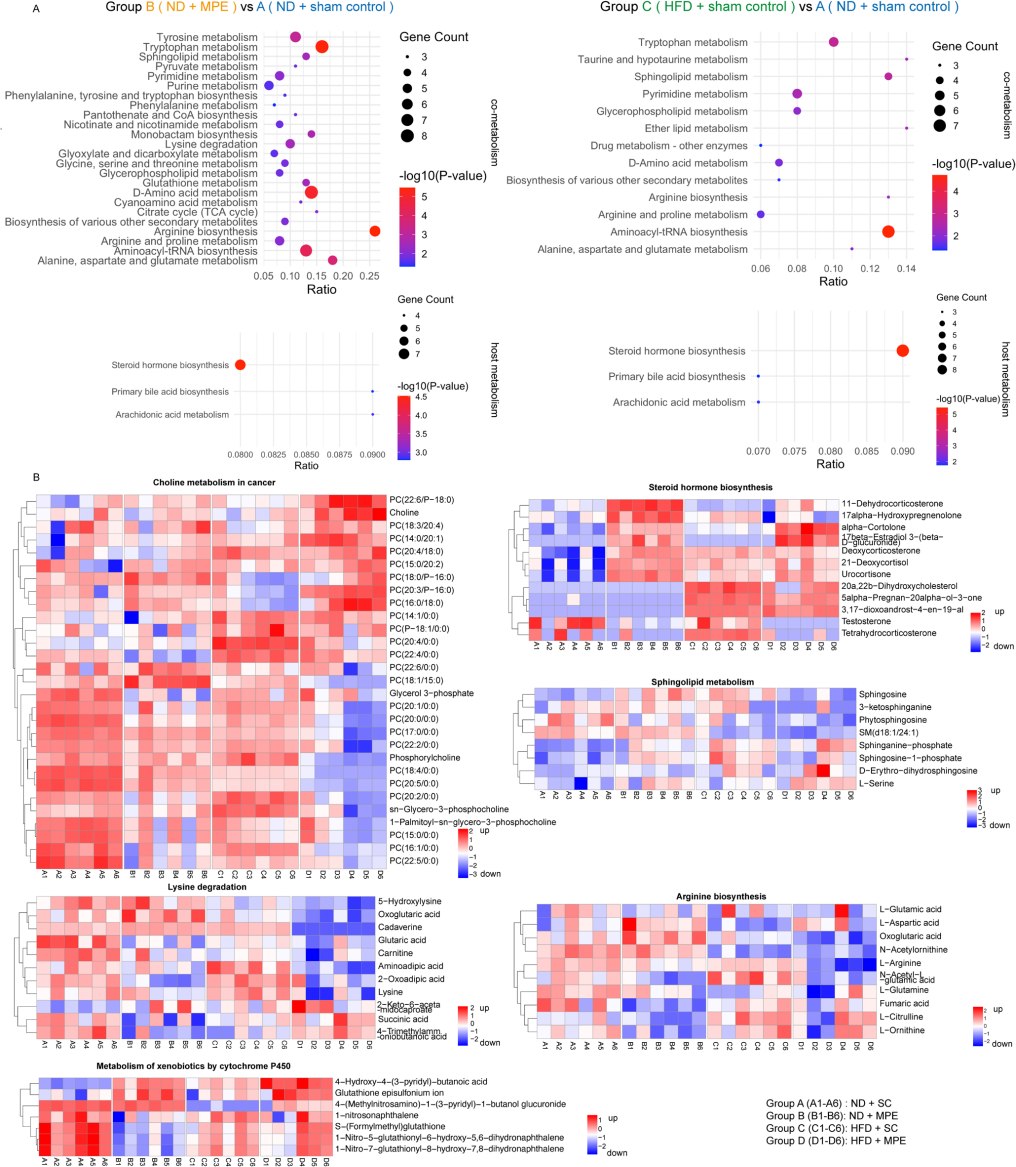
Pathway enrichment and analysis of the serum metabolome. (A) Bubble plot showing the KEGG pathway enrichment analysis of serum metabolites for host metabolism and co‐metabolism performed at Metorigin. Each bubble represents a KEGG pathway, with the *x*‐axis displaying the enriched gene ratio, and the *y*‐axis showing the enriched pathway. The bubble size is proportional to the number of genes involved in the pathway, while the color of the bubble indicates the *p*‐value. (B) Representative heatmaps illustrating the metabolites in the indicated KEGG pathways in each sample. Rows represent the metabolites in the indicated pathway, columns are separated by groups. HFD, high‐fat diet; MPE, malignant pleural effusion; ND, normal diet; SC, sham control.

To understand the metabolomic change in the host and those that co‐exist in the host and intestinal flora, we analyzed KEGG pathway enrichment on the alterations of metabolites from their origin (Figure 6A). We found: In the host levels, both MPE and HFD affect the steroid hormone biosynthesis, primary bile acid biosynthesis, and arachidonic metabolism. In the co‐metabolome, amino acid metabolism, sphingolipid metabolism, glycerophospholipid metabolism, and pyrimidine metabolism were shared in both the altered enrichment caused by MPE and HFD, while MPE causes more alteration including pyruvate metabolism and TCA cycles.

Compared to Group A, Group B showed a downregulation of amino acid metabolism and an upregulation of glucocorticoid biosynthesis, highlighting metabolic shifts under MPE conditions. As for the impacts of high‐fat diet‐induced gut dysbiosis, Group C exhibited an upregulation of steroid metabolism along with a downregulation of cholesterol biosynthesis and xenobiotics metabolism compared to Group A. Then we drew the heatmaps of the metabolites in the 24 samples and clarified the specific change of the single metabolites in the specific sample on the 6 pathways (choline metabolism in cancer, steroid hormone biosynthesis, sphingolipid metabolism, lysine degradation, arginine biosynthesis and metabolism of xenobiotics by cytochrome P450) highlighted in the KEGG and Reactome Enrichment analysis (Figure 6B). The metabolites displayed in the heatmap show distinct expression patterns among groups.

**FIGURE 7:**
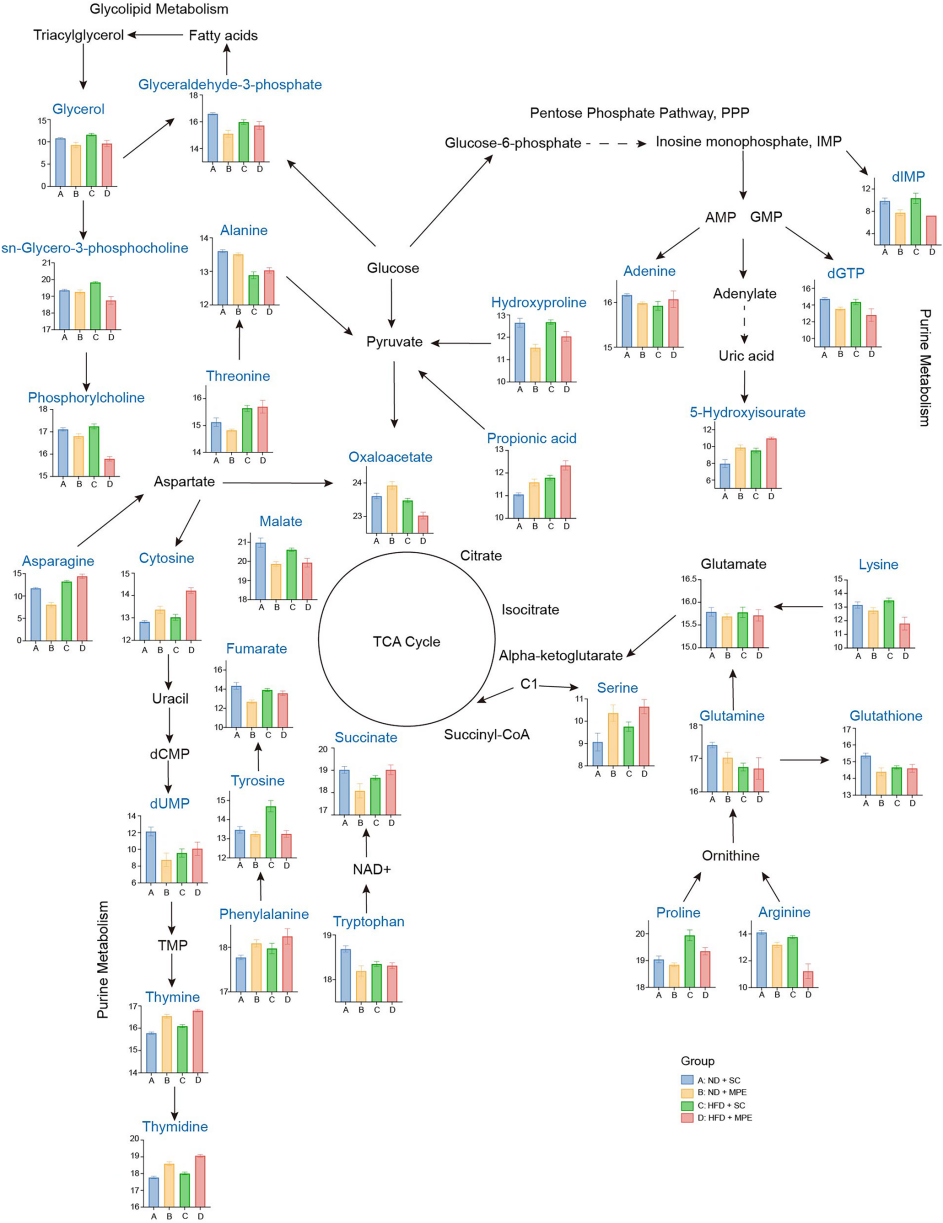
Proposed metabolic pathway from representative altered metabolites in plasma from MPE and HFD mice. In the bar plot, *y*‐axis represents specific metabolites levels after Log_2_ change, while *x*‐axis represents indicated groups. HFD, high‐fat diet; MPE, malignant pleural effusion; ND, normal diet; SC, sham control.

To illustrate representative alteration of metabolites in the plasma under the HFD and/or MPE conditions, a simple metabolic graph with certain metabolite levels was drawn to describe the metabolic pathways showing the potential diet and tumor effects (Figure 7). In this graph, amino acid metabolism, glycolipid metabolism, purine metabolism, and pyrimidine metabolism were connected with the tricarboxylic acid cycle. The direction of metabolite changes in the adjacent metabolites may indicate the potential regulatory relationship.

### 3.4 Muti‐Omics Analysis Based on Metagenome and Metabolome

**FIGURE 8:**
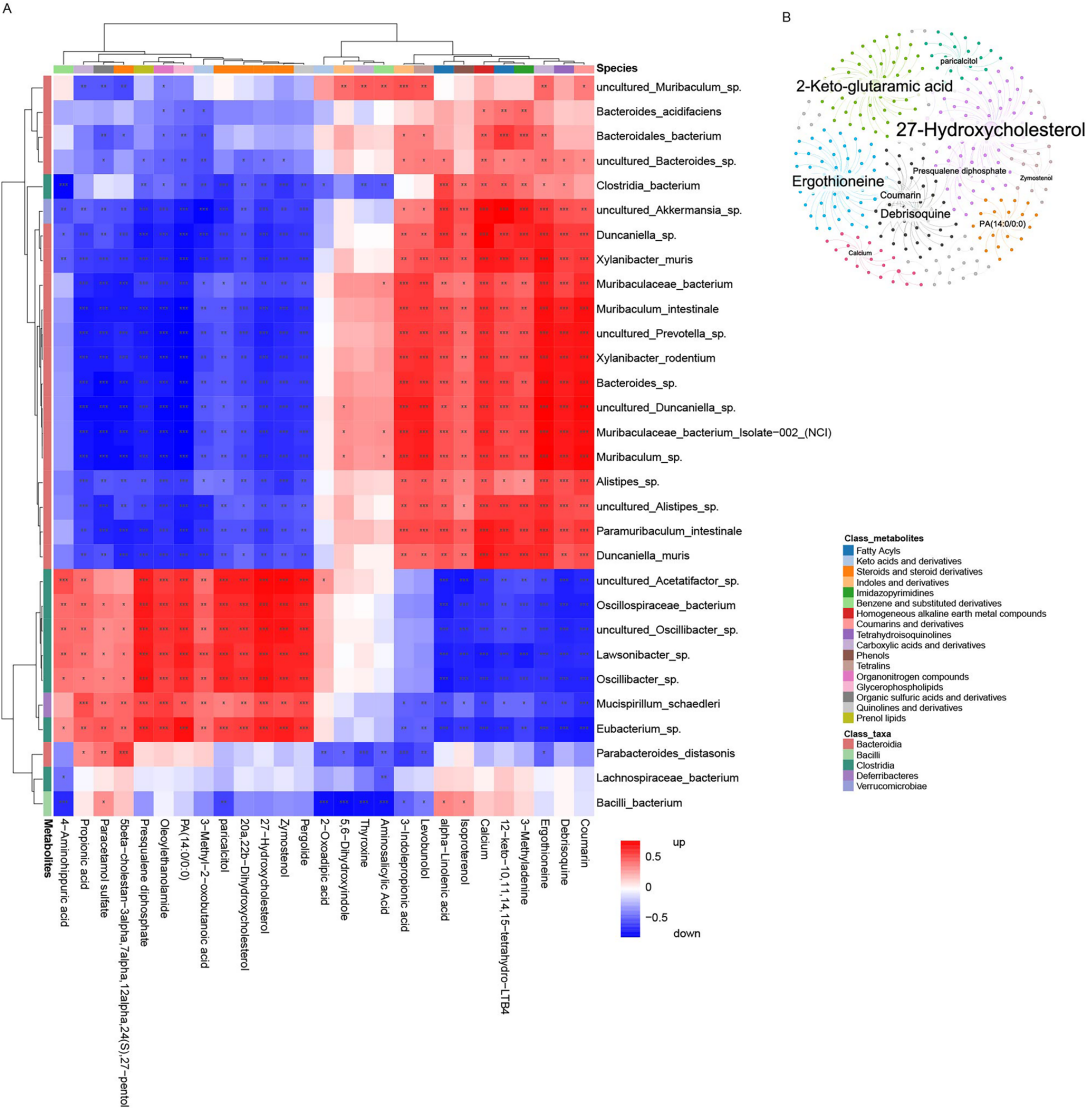
The relationship between the serum metabolites and the gut microbiome. (A) Heatmap illustrating the association between taxa and metabolites and their abundances across different groups. Rows represent taxa at the species level with corresponding class labels indicated. Columns represent metabolites labeled with respective metabolite classes. (B) Gut metagenomics and serum metabolomics correlation network. Nodes represent taxa or metabolite, edges represent significant correlations with Spearman test. (*p* < 0.05, |r| > 0.9).

To elucidate the regulatory relationships between the gut microbiota and metabolite profiles, we performed a correlation network analysis integrating metagenomic and metabolomic data (Figure 8B). Ergothioneine, 27‐hydroxycholesterol, 2‐keto‐glutaramic acid, debrisoquine, coumarin, paricalcitol, zymostenol, calcium, presqualene diphosphate, and PA (14:0/0:0) played essential roles in the influence of the gut microbiome on the plasma metabolome. Heatmaps summarizing the integrated correlations between microbial taxa and metabolites revealed the regulation across groups. For instance, there was a strong positive correlation between LTB4 and *Bacteroida*, suggesting that the downregulation of *Bacteroida* in the intestinal flora, induced by HFD, may lead to a subsequent decrease in LTB4 levels due to the change in host immune response (Figure 8A).

## 4 Discussion

Growing evidence indicates that gut microbiota and their metabolites play a critical role in modulating host immunity and influencing disease progression [11]. However, seldom did the studies focus on the relationship between gut microbiota and the metabolites in MPE development. In our study, we established a dual animal model of HFD and MPE, combining intestinal metagenomic sequencing and plasma metabolomic profiling to examine microbiota–metabolite interactions. Our data revealed that HFD led to a reduced abundance of taxa with a higher *Bacillota*/*Bacteroidota* ratio and a decrease of *Akkermansiaceae*. These alterations were associated with significant changes in lipid, glucose, amino acid, and pyruvate metabolism, highlighting the impact of HFD‐induced dysbiosis on systemic metabolic homeostasis in MPE.

MPE is a disease associated with high hospitalization rates, poor prognosis, and significant healthcare burden [1]. The tumor microenvironment (TME) in MPE, which is made up of cell components, extracellular matrix, and soluble molecules, determines the progress of MPE and the prognosis of the cancer. Cancer cells, immune cells, and stromal cells make up the cell components of TME in MPE [12]. Both the tumor cells and immune cells undergo metabolic reprogramming, consuming shared nutrients such as glucose, glutamine, and arginine, and producing metabolites that modulate immune responses. For example, tumor cells preferentially undergo aerobic glycolysis, producing lactate, while immune cells adjust their metabolic pathway to the acidic environment [13, 14, 15].

HFD‐induced obesity has been associated with the progress of multiple diseases including cancer [16]. Gut microbiota serves as a link between the diet and the host in cancer [17, 18]. Consistent with the previous studies, HFD‐modeled mice exhibited an increased *Bacillota/Bacteroidota* ratio [19]. In response to the MPE, mice on a ND significantly elevated the abundance of the *Akkermansiaceae*, which has been proved as an anti‐tumor factor in NSCLC [20]. *Akkermansiaceae*‐derived a15:0‐i15:0 PE can activate the TLR2–TLR1 signal, leading to an anti‐tumor effect [21]. However, this beneficial response was absent in HFD‐fed mice, suggesting that HFD may impair the gut microbiota's ability to support host immunity during MPE.

*Parabacteroides*, including *Parabacteroides distasonis* and *Parabacteroides goldsteinii* , participate in carbohydrate metabolism and produce short chain fatty acids (SCFAs) [22]. In our study, plasma propionic acid—a major SCFA—was elevated in both HFD and MPE mice, with a cumulative effect in the HFD + MPE group. Propionate has been reported to trigger apoptosis in cancer cells via downregulation of arginine methyltransferase [23]. However, SCFAs serve dual effects on tumor progression: they can affect tumor progression by promoting the T cell differentiation into either effector T cells or Tregs [24]. Meanwhile, *Parabacteroides distasonis* has been reported to stimulate the IL‐10 production in Tregs [25], potentially contributing to immune suppression in the tumor microenvironment. Additionally, *Parabacteroides goldsteinii* produces acetic and succinic acids as the main end‐products of glucose metabolism, which may explain the elevated levels of succinate in the HFD + MPE mice, reflecting HFD‐induced alterations in the TCA cycle [22].

Members of the Muribaculaceae family, such as *Muribaculum intestinale* and *Muribaculaceae* bacterium Isolate‐002 (NCI), are known for their immunostimulatory properties and cross‐feeding relationships with probiotics [26, 27]. These bacteria also harbor genes encoding glycoside hydrolases (GHs) and glycosyltransferases (GTs), which are central to carbohydrate metabolism and cancer‐associated glycosylation patterns [28, 29, 30]. HFD‐induced depletion of *Muribaculaceae* likely contributes to microbial imbalance and impaired immunometabolic functions.

In the host metabolome, both HFD and MPE induced steroid hormone biosynthesis. Obesity‐related hormone disruption can lead to polycystic ovary syndrome [31] and male feminization [32]. In MPE, ectopic hormone production may lead to paraneoplastic syndromes [33]. HFD also alters the bile acid composition, which in turn regulates lipid absorption, gene expression, and gut microbial profiles [34, 35]. The elevation of arachidonic acid—a known promoter of cancer and prognostic marker—further supports the link between lipid metabolism and MPE progression [36, 37, 38, 39].

HFD also activated the PPAR pathway, a key regulator of lipid metabolism. PPARs respond to fatty acids and mediate metabolic reprogramming, contributing to therapeutic resistance in cancer by regulating oxidative stress, apoptosis, and proliferation and immunosuppression [40, 41, 42, 43].

The KEGG pathway enrichment analysis, based on microbiota metagenomic data, aligns with the enrichment results obtained from metabolite profiling, highlighting the functional coordination between microbial and host metabolism. Amino acid metabolism was markedly disrupted, with lower levels of lysine and arginine in the HFD + MPE group, compared to the MPE group. These amino acids are crucial for mitochondrial function, nitric oxide production, and TCA cycle replenishment [44], and their depletion may reflect metabolic competition between tumor and immune cells.

In the metabolism of xenobiotics by cytochrome P450, nitrosamine 4‐(methylnitrosamino)‐1‐(3‐pyridyl)‐1‐butanone (NNK) was downregulated in the HFD group, though it was partially upregulated in the MPE group, consistent with the role of CYP2A13 in its activation in lung cancer [45]. Meanwhile, S‐glutathionylation (SG), a protective modification against oxidative stress, was reduced in MPE mice, aligning with patterns observed in cancer [46].

Altered choline metabolism–a hallmark of many cancers–was also observed [47].

We observed various acquisitions of antibiotic tolerance of HFD, which was thought to be triggered by IAA mainly produced by the *Bacteroides* spp. and *Clostridium* spp. [48].

Despite these promising findings, several limitations should be acknowledged. First, although metagenomic and metabolomic analyses revealed correlations among gut microbiota, plasma metabolites and MPE progression, direct causality has not been established. Second, the MPE mouse model based on a high‐fat diet may not fully reflect the complexity of human pleural malignancies and metabolic diversity. Future studies incorporating microbial transplantation or metabolite intervention, as well as larger cohorts and clinical samples, are needed to validate and expand upon our findings. Comprehensive analysis of metabolites in both pleural effusion and the gut, along with longitudinal sampling at early disease stages and the initiation of HFD exposure, will help to elucidate the dynamic regulatory interactions among the gut microbiota, host metabolism, and MPE progression.

Given the observed role of HFD in promoting MPE progression, maintaining a balanced diet may be important for patients with lung cancer to reduce the risk of MPE development.

## 5 Conclusions

In conclusion, this study revealed key gut microbial taxa and circulating metabolites linked to MPE. Moreover, the study revealed that HFD may accelerate MPE through gut dysbiosis and metabolic alterations, highlighting the potential of dietary intervention and microbiota‐targeted therapies for MPE management.

## Author Contributions

Q.‐Y.C., M.‐M.S., and S.‐F.D. designed the study, performed the experiments, and analyzed the data. F.‐S.Y. and Q.‐Y.C. drafted the manuscript. F.‐S.Y. and H.‐Z.S. conceived the idea, supervised the research, and revised the manuscript. All authors read the manuscript and approved the final version for submission.

## Conflicts of Interest

The authors declare no conflicts of interest.

## Data Availability

The metabolome data generated from this study are available in the NCBI Sequence Read Archive (SRA), under the BioProject accession number PRJNA1211530. The associated BioSample IDs are from SAMN46212320 to SAMN46212343. The sequencing data can be accessed via the SRA database using the following link: https://www.ncbi.nlm.nih.gov/bioproject/PRJNA1211530. The processed results are available from the corresponding author upon reasonable request.
